# Chest CT for Breast Cancer Diagnosis

**DOI:** 10.3390/life12111699

**Published:** 2022-10-26

**Authors:** Elise Desperito, Lawrence Schwartz, Kathleen M. Capaccione, Brian T. Collins, Sachin Jamabawalikar, Boyu Peng, Rebecca Patrizio, Mary M. Salvatore

**Affiliations:** 1 Department of Radiology, Columbia University Irving Medical Center, New York, NY 10032, USA; 2 Department of Radiation Oncology, University of South Florida Tampa General Hospital, Tampa, FL 33612, USA

**Keywords:** breast cancer, chest CT, cancer screening, breast density, mammography

## Abstract

Background: We report the results of our retrospective analysis of the ability of standard chest CT scans to correctly diagnose cancer in the breast. Methods: Four hundred and fifty-three consecutive women with chest CT scans (contrast and non-contrast) preceding mammograms within one year comprise the study population. All chest CT images were reviewed by an experienced fellowship-trained chest radiologist and mammograms by an experienced fellowship-trained mammographer without the benefit of prior or ancillary studies; only four mammographic views were included for analysis. The size, location, and shape of breast masses were documented; on CT, the average Hounsfield units were measured. On both imaging modalities, the presence of lymphadenopathy, architectural distortion, skin thickening, and microcalcifications were recorded. Ultimately, the interpreting radiologist was asked to decide if a biopsy was indicated, and these recommendations were correlated with the patient’s outcome. Findings: Nineteen of four hundred and fifty-three patients had breast cancer at the time of the mammography. Breast masses were the most common finding on chest CT, leading to the recommendation for biopsy. Hounsfield units were the most important feature for discerning benign from malignant masses. CT sensitivity, specificity, and accuracy of CT for breast cancer detection was 84.21%, 99.3%, and 98.68% compared to 78.95%, 93.78%, and 93.16% for four-view mammography. Chest CT scans with or without contrast had similar outcomes for specificity and accuracy, but sensitivity was slightly less without contrast. Chest CT alone, without the benefit of prior exams and patient recall, correctly diagnosed cancer with a *p*-value of <0.0001 compared to mammography with the same limitations. Conclusion: Chest CT accurately diagnosed breast cancer with few false positives and negatives and did so without the need for patient recall for additional imaging.

## 1. Introduction

Breast cancer is a leading cause of morbidity and premature death for women. Over 300,000 women will be diagnosed with breast cancer this year and the number is increasing as the population ages [1,2]. Imaging of breast cancer is not a new concept; Robert Eagan’s 1963 publication showed that mammography had a 79% sensitivity for breast cancer diagnosis, which provided the foundation for mammography as a screening tool [3]. The Cochrane review stated that screening reduces breast cancer mortality by 15%, but at a cost of over-diagnosis with resultant over-treatment of 30%, especially for ductal carcinoma in situ (DCIS) [4]. An alternative screening tool is needed that is more sensitive and specific. Recent advances in three-dimensional chest CT with dose-lowering technology and improved image resolution could make it a promising contender.

CT scans were first developed in the 1970s [5]. The breasts were imaged on these first-generation scanners, but information regarding the breast parenchyma was limited and largely ignored. In the 1990s, newer-generation spiral CT scanners allowed rapid image acquisition and reconstruction without additional radiation exposure allowing advances in the diagnosis of pulmonary emboli, coronary artery calcifications [6], and aortic disease [7]. The breast tissue is imaged on chest CT with no added radiation [8]. The idea of a breast cancer diagnosis on chest CT may be analogous to lung cancer screening with chest CT; Henschke reported that non-calcified nodules were detected in 23% of participants by CT compared with 7% by chest radiography. CT diagnosed lung cancer in 2.7%, while chest X-ray diagnosed only 0.7% [9]. The three-dimensional capability of CT was superior to the two-dimensional chest X-ray for lung cancer detection, and so it follows that it may be valuable for the detection of breast cancer.

Our earlier work confirmed chest CT’s ability to evaluate breast density; patients with higher breast density are at greater risk for developing breast cancer [10,11]. Our inter-reader agreement for radiologists with expertise in mammography was higher for CT breast density than for mammographic breast density (kappa value: 0.79 versus 0.62) [11]. A reader study consisting of seven general radiologists evaluating breast density on chest CT demonstrated substantial to excellent agreement with the expert consensus grading (kappa value: 0.61–0.88) [10]. In our third study, eighty-five masses were identified on 542 CT scans. Many of them had Hounsfield units (HUs), less than 20 representing cysts on CT obviating the need for ultrasound. Eleven invasive cancers were seen on mammography and nine (82%) on chest CT. For the two cancers not identified on CT, the CT scans were obtained seven months and four months before the mammogram [12]. The false-positive rate of chest CT in that study was 5%, which compares favorably to the reported 7–12% false-positive rate of mammography [13], considered a significant drawback of the modality [14]. There was excellent agreement between CT and mammography for normal (88%) and the overall agreement was 77% [12]. The purpose of this study is to build upon our prior research by performing a head-to-head comparison, devoid of benefits provided by prior and ancillary imaging, to directly compare the sensitivity, specificity, and accuracy of the two modalities for breast cancer diagnosis.

## 2. Methods

IRB approval was obtained for this retrospective, HIPAA-compliant study (IRB-AAAS3915). The need for informed consent was waived for this retrospective study. The hospital’s imaging database was queried for sequential adult female patients (21 years or older) who had a standard chest CT with or without contrast preceding a mammogram (screening or diagnostic) by no more than 365 days and no intervening biopsy. Four hundred and fifty-three patients met inclusion criteria.

Chest CT scans (with and without contrast) were performed using four GE 64-slice CT scanners (two Revolution, one Discovery 750 HD, and one Optima 660, GE Healthcare, Milwaukee, WI, USA) and one 16-slice CT scanner (Optima 540, GE Healthcare, Milwaukee, WI, USA) at our site. The unified acquisition parameters for 64-slice CT were as follows: tube voltage 120 kVp, tube current modulation (range 80–600 mA), Noise index 16, pitch 1.375, and collimation 40 mm. The 16-slice CT used the same settings, except for pitch 0.938, and collimation 20 mm. For all the scans, two axial reconstructions were generated: one using adaptive statistical iterative reconstruction (ASIR) level of 40%, 1.25 mm slice thickness, and standard kernel; the other using ASIR 20%, 5 mm slice thickness, and lung kernel. Contrast studies were performed using approximately 100 ccs of intravenous non-ionic contrast agent Omnipaque 350. Chest CT images were reviewed in axial projection on soft-tissue window settings using available collimation (1 to 5 mm). The mean effective doses for standard chest CT and low dose CT at our site are 5.8 mSv and 0.51 mSv, respectively.

A fellowship-trained thoracic radiologist with over 20 years of experience reviewed the chest CT images without the benefit of any prior or ancillary studies. Note was made if the chest CT was with or without contrast and if the entire breast was included on the image. The extent of breast fibroglandular tissue was noted (Grade1; 0–25% fibroglandular tissue; Grade 2: 26–50% fibroglandular tissue; Grade 3: 51–75% fibroglandular tissue, and Grade 4: >75% fibroglandular tissue). Breast masses were evaluated for size and border configuration. The average Hounsfield unit of a mass was measured. The presence of lymphadenopathy, architectural distortion, skin thickening, and calcifications were documented. Ultimately, the radiologist was asked to decide if the patient needed a biopsy and this result was correlated with the ground truth derived from a review of the medical record including results from prior imaging, ultrasound, MRI, biopsy when available, and clinical follow-up for at least one year.

Mammograms were performed on dedicated mammography units (Senographe Essential, GE Healthcare, Milwaukee, WI, USA). The views obtained consisted of the standard mediolateral oblique and craniocaudal views. For image acquisition, the parameters were as follows: kVp 29, mAs 35–77, and the mean effective dose of 0.72 mSv for all four views.

A fellowship-trained breast imager with over twenty years of experience reviewed the mammogram without the benefit of any prior or ancillary studies; only four standard mammographic views were analyzed. The extent of breast fibroglandular tissue was noted. Breast masses were evaluated for size and border configuration. The presence of lymphadenopathy, architectural distortion, skin thickening, and calcifications were documented. Ultimately, the radiologist was asked to decide if the patient needed a biopsy and this result was correlated with the ground truth derived from a review of the medical record including results from prior imaging, ultrasound, MRI, biopsy when available, and clinical follow-up for at least one year. True negative (TN) was defined as no cancer and CT scan and/or mammogram interpreted as negative; true positive (TP) was a patient with cancer and CT scan and or mammogram interpreted as positive; false negative (FN) was a patient with cancer, and CT scan and/or mammogram interpreted as negative; false positive (FP) was a patient without cancer and CT scan and/or mammogram interpreted as positive. The data that support the findings of this study are available from the corresponding author, [MS], upon reasonable request.

## 3. Findings

**Figure 1:**
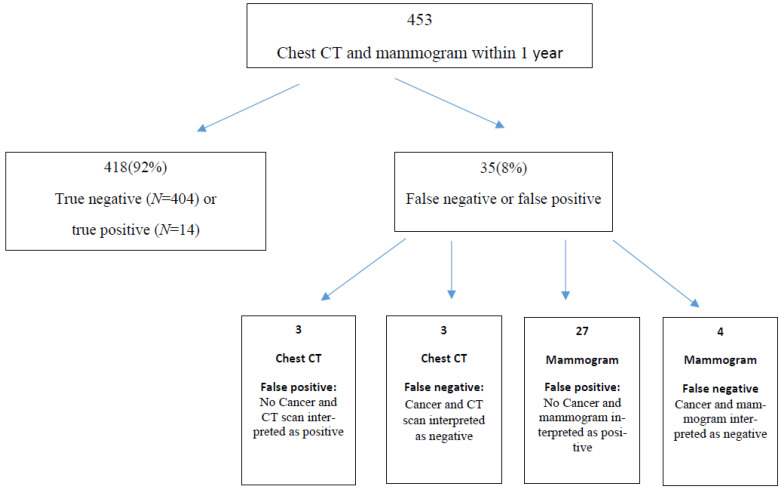
Results of imaging analysis. True negative (TN): No cancer, and CT scan and/or mammogram interpreted as negative; true positive (TP): cancer, and CT scan and/or mammogram interpreted as positive; false negative (FN): cancer, and CT scan and/or mammogram interpreted as negative; false positive (FP): no cancer, and CT scan and/or mammogram interpreted as positive.

Four hundred and fifty-three female patients over the age of 21 were included in our study. For four hundred and eighteen patients, both exams were either true negative or true positive. For the remaining 35 patients, one or both exams were false positive or false negative (Figure 1).

### 3.1. Overview

The average age for 434 patients without cancer was 65 years (range 31–92 years). The average age for 19 patients diagnosed with breast cancer was 62 years (range 29–86 years). Sixteen chest CT scans and mammograms were performed on the same day. The average difference in time between the two imaging modalities was 126 days (range 0 to 365 days) with the chest CT scan preceding the mammogram for all patients. In total, 171 CT scans were with intravenous contrast, the remaining 282 were without. Both breasts were imaged in their entirety for 303 patients. Ten patients had a prior right mastectomy and eight patients had a prior left mastectomy leaving only one breast to be examined on imaging.

Nineteen of four hundred and fifty-three patients had cancer of the breast at the time of mammography. Twelve were invasive ductal cancer, one was invasive lobular cancer, one DCIS, one leukemia, one lymphoma, and three metastatic disease. The sensitivity, specificity, and accuracy of CT for breast cancer detection were 84.21%, 99.3%, and 98.68% compared to 78.95%, 93.78%, and 93.16% for mammography (Table 1). Chest CT alone, without the benefit of prior exams or patient recall, correctly diagnosed cancer in the breast with a *p*-value of <0.0001 compared to mammography with the same limitations. The specificity and accuracy were essentially the same for contrast and non-contrast chest CT scans. The sensitivity of chest CT was better with contrast (85.71% vs. 80.00%) (Table 2). The sensitivity of chest CT without contrast was slightly better than mammography (80.00% vs. 78.95%).

### 3.2. Breast Density

The extent of breast fibroglandular tissue by visual inspection was higher on average on mammography than on CT (2.42 vs. 2.23) with *p* = 0.0004 and more people were assigned a breast density grade of 3 to 4 on mammogram (*p* = 0.04). CT had low breast density in 96% of normal mammograms and high breast density in 5% with cancer. Mammography had low breast density in 95% of normal mammograms and high breast density in 3% with cancer (Table 3).

### 3.3. Masses

**Figure 2:**
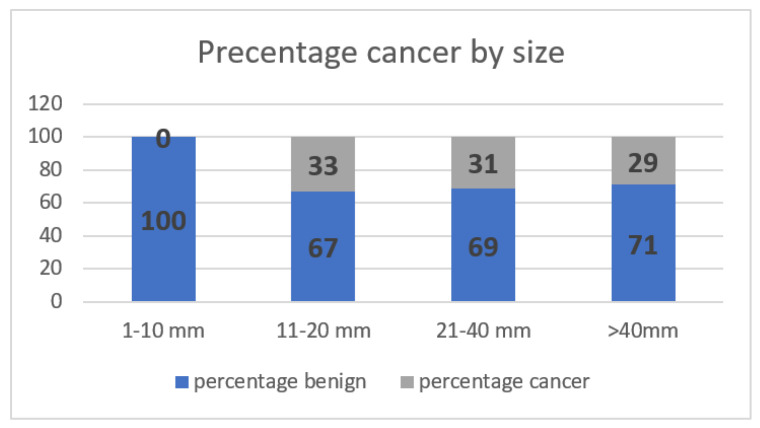
Percentage of breast cancers based on the size of breast mass on chest CT.

**Figure 3:**
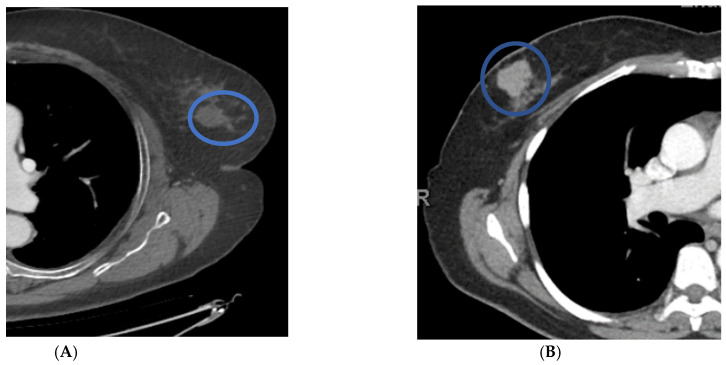
Distinguishing features of benign versus malignant masses on contrast-enhanced chest CT scans in axial projection. Patient (**A**) has a benign nodule with smooth borders and HUs of less than twenty. Patient (**B**) has a malignant nodule with lobular borders and HUs greater than twenty.

Forty-nine patients had masses on mammograms, of which 16% were cancer, and there were forty-four masses identified on CT, of which 20% were cancer. On chest CT, the average size of a cancerous mass was larger; no nodules less than 10 mm were cancer (*p* = 0.06) (Table 4) (Figure 2). The average HU of cancerous nodules was higher; no cancer had a HU of less than twenty-four (*p* = 0.003). Cancerous nodules were often lobular or spiculated (Figure 3). All nine cancerous nodules on CT were recommended for biopsy and two of thirty-five benign nodules were recommended for biopsy.

### 3.4. Calcifications

**Figure 4:**
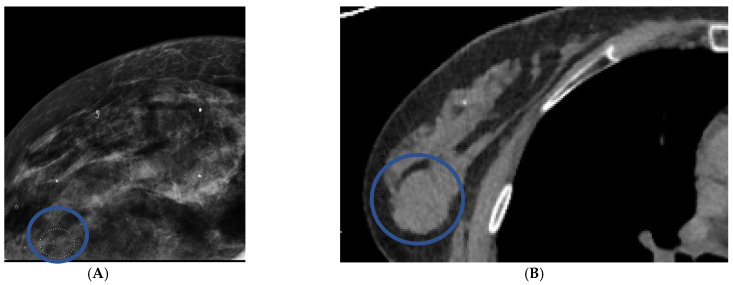
Right upper outer quadrant microcalcifications on craniocaudal mammography were recommended for biopsy (**A**). Axial chest CT performed 128 days prior to the mammogram recommended biopsy for a 20 mm right upper outer quadrant mass (**B**). Biopsy results at the time of mammography were ductal carcinoma in situ.

**Figure 5:**
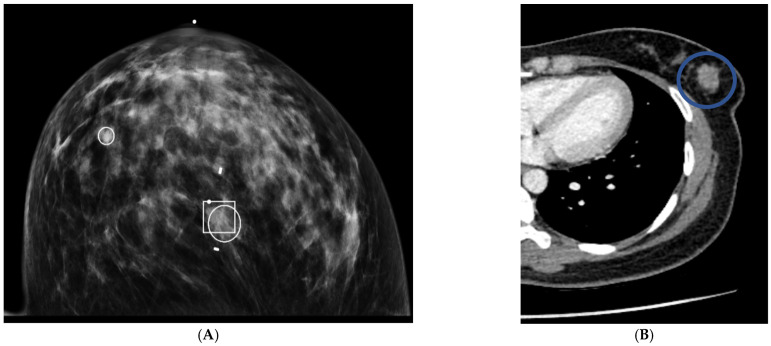
Craniocaudal mammographic image reveals clustered left breast microcalcifications that were recommended for biopsy (**A**). Contrast chest CT in axial projection on the same day reveals a 20 mm mass with an average HU of 61 (**B**). Biopsy results were invasive breast cancer.

Microcalcifications were identified on twenty mammograms, and biopsy was recommended in five (25%). In total, 2/20 (10%) patients with micro-calcifications on mammography had breast cancer. No micro-calcifications were identified on CT. In patients who had micro-calcifications on mammography, CT correctly diagnosed two true positive nodules (Figure 4 and Figure 5), one of which was ductal carcinoma in situ and the other was invasive breast cancer. The remaining eighteen patients with microcalcifications on mammography were correctly identified as normal on chest CT.

### 3.5. Findings Associated with Cancer

Adenopathy was an infrequent finding on mammography (1.3%); however, 67% were associated with cancer. In contrast, adenopathy was visualized frequently on chest CT (5.5%), but only associated with cancer in 36%. Skin thickening was commonly reported on mammography and associated with cancer in 4/44 (9%); in contrast, CT demonstrated skin thickening associated with cancer in 4/13 (31%). Architectural distortion was reported on mammography 93 times with a 4% association with cancer. Similarly, 3% of patients with architectural distortion on CT had cancer (Table 5).

## 4. Discussion

Our focus will be on chest CT findings in the breast, as the attributes of mammography for breast cancer diagnosis have been extensively reported in the literature. Our results exceeded expectations for the sensitivity, specificity, and accuracy of chest CT for breast cancer diagnosis. The true negative and true positive rate for both modalities was 92% in the current study, similar to our prior results [12]. Mammography and chest CT had comparable false-negative rates, but mammography had many more false-positive exams, which may be attributed to the 3-dimensional nature of CT that removes overlapping tissues, which allowed chest CT to recommend half as many biopsies (20 vs. 44) with no patient recall. This could be most helpful in patients with dense breast parenchyma who are at the greatest risk for developing breast cancer. When we consider patients who are characterized as having a grade of 3 or 4 on mammography for the extent of fibroglandular tissue, there were twelve false positives on mammography and none on chest CT. CT with and without contrast had similar specificity and accuracy, but the use of contrast increased sensitivity slightly, which is expected. Both breasts were imaged in their entirety on 321 patients. The lack of inclusion of the entire breast did not interfere with a diagnosis for the 132 patients, but it is an obvious limitation if viewing techniques are not adjusted to include all scanned tissue [8].

**Figure 6:**
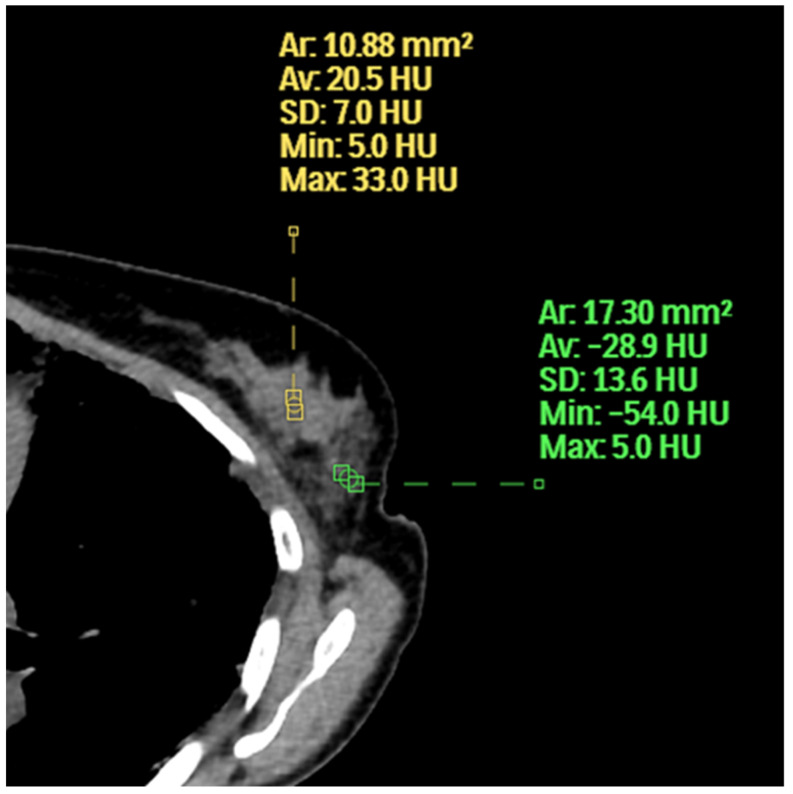
The inner aspect of the left breast has a higher average HU and maximum HU than the outer aspect.

For this paper, we used the term extent of fibroglandular tissue instead of breast density because, on chest CT, one can see that all hyperdense areas relative to fat are not the same. There are higher and lower areas of breast density as measured by HU (Figure 6). The HU is a measure of the absorption/attenuation coefficient of radiation on CT and the density of tissue is proportional to the measurement [15]. The average breast density on mammography was higher than chest CT because mammography underestimates the amount of fat due to overlapping soft tissue. Perhaps not surprisingly, many cancers occurred in patients with low breast density because the occurrence of cancer is increased by not only the extent of the disease but by years of exposure. This topic goes beyond the scope of our manuscript but is well described in Boyd’s calculation of the cumulative percent mammographic density which considers age [16].

Masses were the most common finding on chest CT scans, prompting a recommendation for biopsy (12/20 (60%)). The cancerous masses were, on average, larger than non-cancerous masses and with higher Hounsfield units, as would be expected, as nodules with HUs less than 20 are often cysts. The majority of the cancerous masses were spiculated or lobular. The CT evaluation of HU and border contributed to the correct diagnosis for 33/35 benign masses and 9/9 cancerous. These results are promising but further research will determine if HUs could replace ultrasound exams for breast cancer evaluation.

We were concerned about the inability of CT scan to recognize micro-calcifications. Twenty patients had microcalcifications on mammography, of which two were cancer. CT correctly diagnosed these two patients because a nodule was seen on CT in the area of microcalcifications. A nodule was not identified on mammography for these two patients. The findings suggest that, like MRI, CT may diagnose the most clinically relevant microcalcifications, but further investigation is needed [17].

Adenopathy was identified more frequently on chest CT because of its ability to image the entire axilla. Skin thickening was more frequently diagnosed on mammography but more frequently associated with cancer on chest CT. Architectural distortion was much less frequent on CT because of 3-D capability and, therefore, more specific for breast cancer.

Chest CT for breast cancer screening meets the required criteria for a screening exam [18]. It has high sensitivity and specificity. The average effective dose from one lung cancer screening CT is less than 1.0 mSv [19] compared to a standard four-view digital mammography with an average effective dose of 0.68 mSv [20]. Many patients have additional magnification and/or spot compression images or tomosynthesis for the workup of an abnormality. The 2017 Medicare reimbursement costs for a screening mammogram and chest CT are comparable [21,22]. 

**Figure 7:**
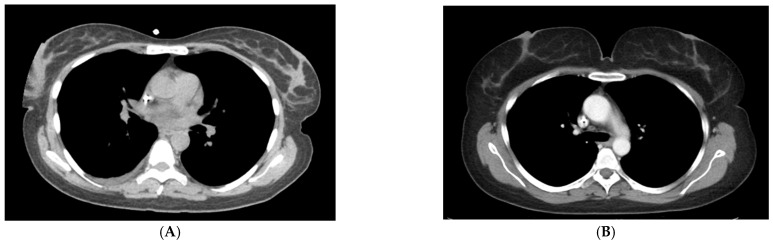
Same patient without and then with bra in place. In the first image (**A**), a portion of the right breast is not imaged due to collimation. In the second image (**B**), both breasts are positioned in the area of reduced current due to the wearing of a bra during the CT scan.

There are opportunities to improve upon results achieved in this retrospective study by incorporating skin markers of surgical scars and palpable abnormalities. Requesting patients wear their bras during imaging positions the breasts in a reproducible manner (Figure 7). Generating one series on a CT scan with a field of view that allows visualization of the entire breast should be mandatory. Recently, *Nature* published an article regarding the use of artificial intelligence in mammography and showed a decrease in false positives by 5.7% and false negatives of 9.4% [23]. AI has the potential to differentiate benign from malignant breast masses on CT. Prospective studies are needed to determine if CT can maintain its high sensitivity and specificity for both prevalent and incident breast cancers.

This retrospective study has limitations. The readers in our study have considerable experience and it remains to be seen if less experienced radiologists could obtain similar results. Bias exists in a retrospective study with more people having breast cancer than in a screening population. We were concerned that contrast would provide an added advantage to CT, but we did not find that to be the case; prospective screening studies would be without contrast. The imaging techniques were variable, and the breasts were incompletely imaged on some patients, but this did not seem to affect the results. In addition, the majority of exams were standard dose and further investigation using a low-dose technique will be necessary.

## 5. Conclusions

The results of this original research study of chest CT scans for breast cancer diagnosis are quite remarkable and an imaging exam that allows such high sensitivity, specificity, and accuracy should be investigated prospectively.

## Figures and Tables

**Table 1:** Sensitivity, specificity, and accuracy of mammography compared to chest CT.

	Mammogram	Chest CT
No cancer and exam negative	407	431
Cancer and exam positive	15	16
Cancer and exam negative	4	3
No cancer and exam positive	27	3
Total patients	453	453
Sensitivity	78.95	84.21
Specificity	93.78	99.30
Accuracy	93.16	98.68

**Table 2:** Sensitivity, specificity, and accuracy of chest CT with or without contrast.

	With Contrast	Without Contrast	Both
No cancer and exam negative	155	276	431
Cancer and exam positive	12	4	16
Cancer and exam negative	2	1	3
No cancer and exam positive	2	1	3
Total	171	282	453
Sensitivity	85.71	80.00	84.21
Specificity	98.73	99.64	99.30
Accuracy	97.66	99.30	98.68

**Table 3:** Summary of imaging observations on mammography and chest CT in association with extent of breast fibroglandular tissue.

	Mammogram	CT	*p*-Value
Average breast density	2.42 (range 1–4)	2.23 (range 1–4)	0.0004
% with dense breast tissue (Grade 3 or 4)	182/453 (40%)	152/453 (34%)	0.04
Low breast density and normal	258/271 (95%)	290/301 (96%)	0.50
High breast density and cancer	5/182 (3%)	8/152 (5%)	0.24

**Table 4:** Distinguishing features of benign and malignant masses on chest CT.

	Cancer Mass	Benign Mass	*p*-Value
Number of masses	9	35	0.00006
Average size	34 mm (15–109 mm)	20 mm (4–65 mm)	0.06
Average HU (with/without)	49 HU (55/30)	22HU (22/22)	0.003
Smooth border	33%	93%	0.0001
Lobular border	33%	5%	0.02
Spiculated border	33%	2%	0.004
Biopsy recommended	100%	5%	∞

**Table 5:** Findings associated with breast cancer on mammography and chest CT.

	Prevalence on Mammography	Association of Finding with Cancer	Prevalence on Chest CT	Association of Finding with Cancer	*p*-Value
Micro-calcifications	20/453 (4.4%)	2/20 (10%)	0/453 (0%)	0/0 (0%)	∞
Adenopathy	6/453 (1.3%)	4/6 (67%)	25/453 (5.5%)	9/25 (36%)	0.18
Skin thickening	44/453 (9.7%)	4/44 (9%)	13/453 (2.9%)	4/13 (31%)	0.05
Architectural distortion	93/453 (21%)	4/93 (4%)	15/453 (3%)	0/15 (0%)	∞
Masses	49/453 (11%)	8/49 (16%)	44/453 (10%)	9/44 (20%)	0.61
Biopsies recommended	44/453 (9.7%)	15/44 (34%)	20/453 (4.4%)	17/20 (85%)	0.002

## Data Availability

The data that support the findings of this study are available from the corresponding author, [M.M.S.], upon reasonable request.
